# A case of intra‐abdominal gossypiboma diagnosed preoperatively by endoscopic ultrasound fine‐needle‐aspiration

**DOI:** 10.1002/deo2.388

**Published:** 2024-05-29

**Authors:** Katsuyuki Dainaka, Yoshida Juichiro, Yutaka Inada, Akifumi Fukui, Takeshi Nishimura, Hideki Fujii, Naoya Tomatsuri, Yusuke Okuyama, Hideki Sato, Yoji Urata

**Affiliations:** ^1^ Department of Gastroenterology and Hepatology Japanese Red Cross Society Kyoto Daiichi Hospital Kyoto Japan; ^2^ Department of Pathology Japanese Red Cross Society Kyoto Daiichi Hospital Kyoto Japan

**Keywords:** endoscopic ultrasound fine‐needle‐aspiration, gossypiboma, polarized light microscope, pseudosubmucosal tumor, retained surgical sponge

## Abstract

Gossypiboma is an extremely rare adverse event occurring post‐surgery, where surgical gauze is left within the body. If aseptically retained, it can lead to the formation of granulation tissue through chronic inflammation and adhesion with surrounding tissues, potentially persisting asymptomatically for many years. While diagnosis of this condition has been reported through various imaging modalities such as abdominal ultrasound and computed tomography, cases not presenting with typical findings are difficult for preoperative diagnosis, and instances where it is discovered postoperatively exist. Particularly when in contact with the gastrointestinal tract within the abdominal cavity, differentiation from submucosal tumors of the digestive tract becomes problematic. This report describes the imaging characteristics of endoscopic ultrasound and the usefulness of endoscopic ultrasound‐fine‐needle‐aspiration for tissue diagnosis in the preoperative diagnosis of intra‐abdominal gossypiboma.

## INTRODUCTION

Gossypiboma, a term used for the retained surgical sponge, is a rare complication of surgery, occurring most commonly after abdominal surgery. Studies have reported that gossypiboma can be detected early or several decades after surgery until the disease remains asymptomatic for an extended period. Herein, we report a case of intra‐abdominal gossypiboma that had been asymptomatic for 44 years after surgery and was diagnosed through endoscopic ultrasound fine‐needle aspiration (EUS‐FNA).

## CASE REPORT

**FIGURE 1:**
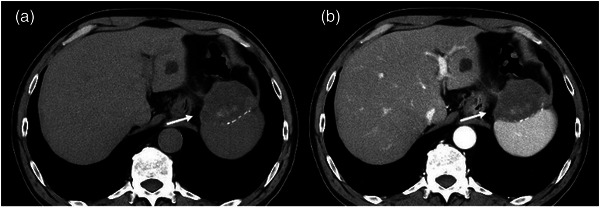
Plain computed tomography (a) showing calcification at the margins and inside of the mass. No clear contrast enhancement effect was observed on contrast‐enhanced computed tomography (b).

**FIGURE 2:**
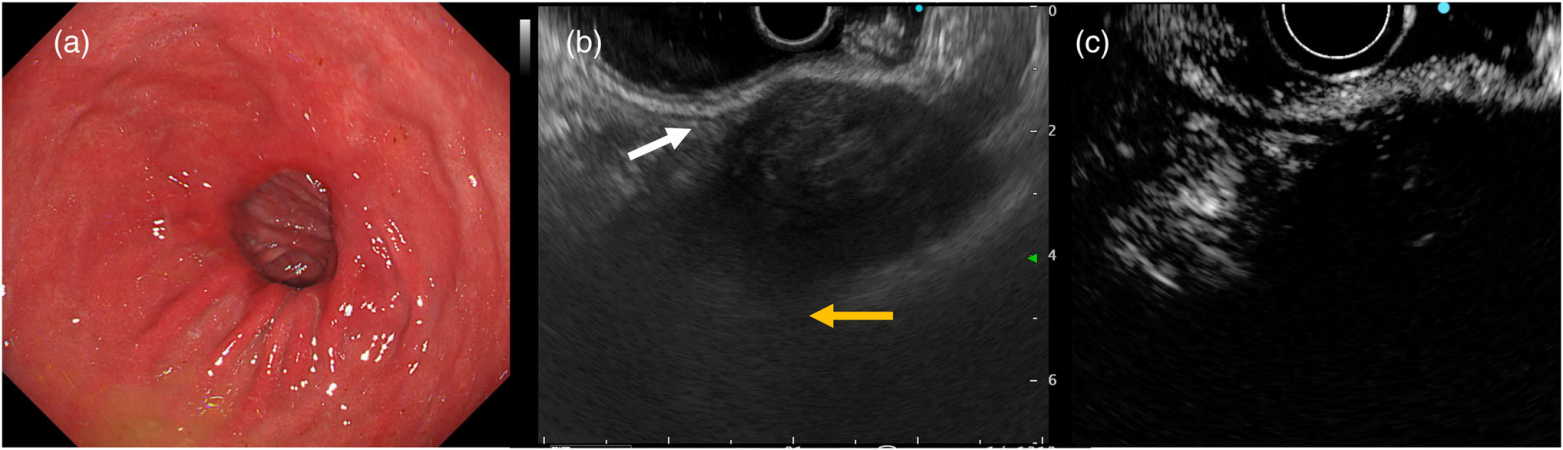
White light imaging (a): After pyloric gastrectomy and Billroth 1 method reconstruction. There was no obvious mass in the gastric lumen; Endoscopic ultrasound (b): Post‐acoustic shadow with low‐echoic mass. Post‐acoustic shadow (yellow arrow); Tumors appear to be in continuity with the gastric wall (white arrow); Contrast‐enhanced endoscopic ultrasound (c): No influx of perflubutane into the mass.

**FIGURE 3:**
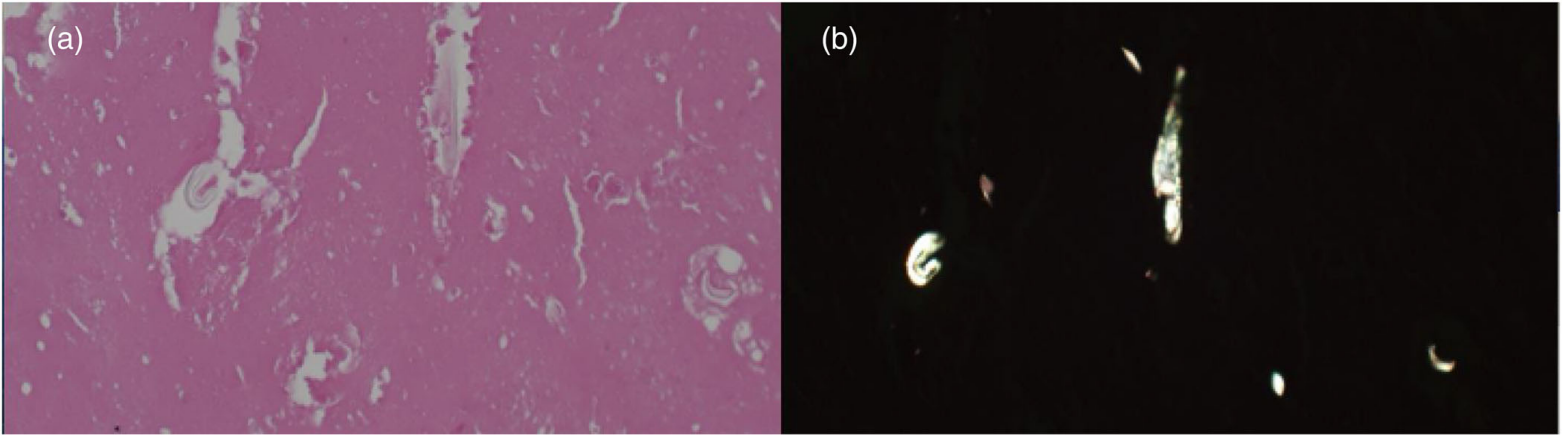
A string of gauze with necrotic tissue was obtained using a 19‐gauge needle. Hematoxylin and eosin stain, ×100 (a); Polarized illumination. Polarized light microscopy was used to highlight the cotton fiber (b).

**FIGURE 4:**
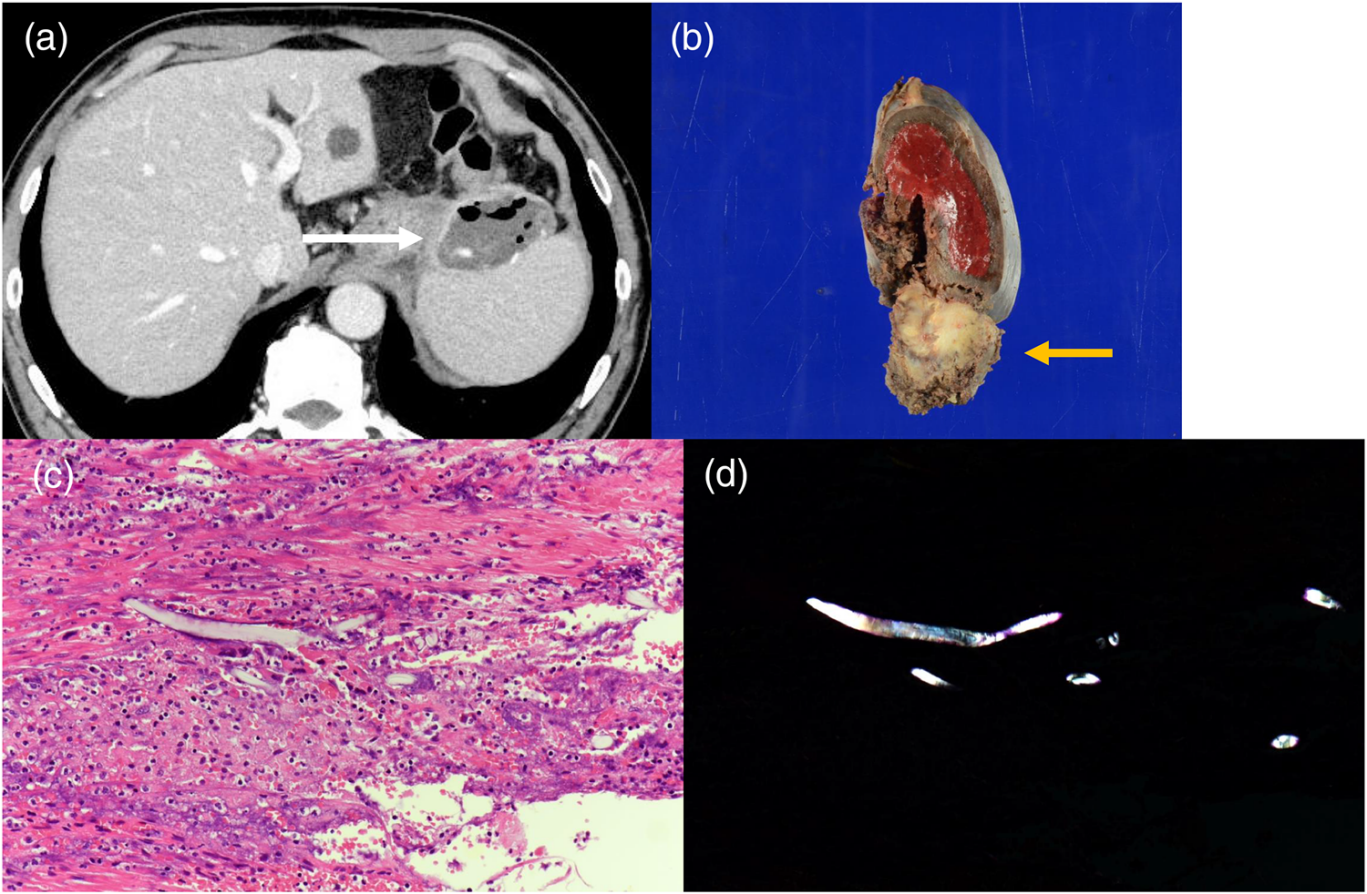
Computed tomography at 1 month after endoscopic ultrasound fine‐needle aspiration. Computed tomography scan after endoscopic ultrasound fine‐needle aspiration reveals the presence of gas within the mass (white arrow). (a) Resected specimen; (b) Hematoxylin and eosin stained specimen (×100). The postoperative specimen reveals a fragmented filamentous foreign body within the granulation tissue with hyalinization and ossification; (c) Polarized illumination. Polarized light microscopy was used to highlight the cotton fiber (d).

A 63‐year‐old male patient, who had undergone distal gastrectomy for gastric ulcer at the age of 19 years, was admitted to the hospital for essential thrombocytopenia. An abdominal ultrasound examination revealed a mass in the upper part of the gastric body, in contact with the spleen. Upper gastrointestinal endoscopy revealed no neoplastic lesions. Abdominal contrast‐enhanced computed tomography (CT) revealed a mass measuring 67.7 × 52.5 mm in contact with the lateral area of the liver, the upper part of the gastric body, and the spleen. The mass exhibited predominantly low absorption internally, accompanied by some surrounding calcification. Additionally, the iodinated contrast agent demonstrated a poor contrast effect, suggesting an ischemic mass. Radiological findings indicated the possibility of gastrointestinal stromal tumor extending outside the gastric wall (Figure 1). Consequently, an ultrasound endoscopy (GF‐UCT260/ EU‐ME2; Olympus Optical Co.) was performed along with an EUS examination, which revealed a 50‐mm mass in contact with the posterior wall of the upper gastric body, protruding outside the wall. Internally, the mass appeared heterogeneous and hypoechoic, with some anechoic areas; when observed underwater, it was partially continuous with the muscularis propria of the stomach. A post‐acoustic shadow was observed from the mass border. Although there were areas that made visualizing the deep part of the mass difficult, we believe that this finding was due to the surrounding calcification noted on CT scans. Contrast imaging with perflubutane revealed only a faint contrast in the marginal parenchyma, with no obvious bubble inflow into the interior (Figure 2). Considering the submucosal mass with heterogeneous internal characteristics originating from the muscularis propria, a highly malignant gastrointestinal stromal tumor was considered a differential diagnosis. To confirm the diagnosis histologically, three transgastric punctures were made using a 19‐gauge Franseen needle (SonoTip TopGain; Medico's Hirata Inc.). In the first and second attempts, we were unable to obtain a sufficient amount of specimen using the dry suction method; however, in the third attempt, we obtained a brittle grayish‐white specimen using the slow‐pull method. Histopathological findings revealed predominantly necrotic tissue, comprising a fibrous interstitium with hyalinization; cotton fibers were confirmed using polarized illumination, leading to a diagnosis of gossypiboma (Figure 3). Contrast‐enhanced CT performed 1 month after FNA revealed a gas image inside the mass, which had reduced to 66.7 × 36.1 mm. This reduction could be attributed to a decrease in fluid content resulting from fine‐needle aspiration and air intrusion into the mass. Splenectomy and tumor removal surgery were performed 3 months after FNA, and cotton fibers were found in the granulation tissue, similar to the FNA specimen (Figure 4).

## DISCUSSION

Gossypiboma is an extremely rare adverse event after surgery, with recent reports suggesting that it occurs in one in 5000–10,000 surgeries.^1^ Early detection of the mass is possible only if a strong inflammatory reaction, such as abscess formation, occurs. However, cases have been reported in which the disease progresses asymptomatically for an extended period, as seen in the present case, where the disease progresses under aseptic conditions and adheres to surrounding tissue due to chronic inflammation to form granulation tissue.^2^ However, even in asymptomatic cases, gossypiboma can cause intestinal obstruction and infectious abscesses, with a reported mortality rate of 75% for abscesses due to secondary infection; hence, surgical removal is recommended after diagnosis.^3^

Studies have reported that ultrasound and CT can be used for preoperative diagnosis. The ultrasound findings of gossypiboma reported by Manzella et al. describe the mass as containing clearly demarcated wavy internal echoes with a hypoechoic ring and strong posterior acoustic shadow,^4^ which is consistent with the EUS findings of this case. No studies have reported on the use of perflubutane for imaging gossypiboma; however, considering the histopathological findings of the postoperative specimen in our case, the contrasted area at the margin of the mass was inferred to be granulation tissue formed around the retained material observed in the postoperative specimen. The CT scan typically reveals a whirl‐like spongiform pattern with gas bubbles, and external high‐density walls, which are more prominent than observed with contrast‐enhanced imaging; these are considered typical findings and have been reported to be accurate for diagnosis.^5^ However, although CT examination in the present case revealed calcification within and around the mass, the typical findings described above were not observed, and a preoperative diagnosis could not be made. Indeed, similar cases have been reported previously where typical imaging findings were not obtained, making preoperative diagnosis based on imaging findings alone challenging.^6^, ^7^, ^8^ A magnetic resonance imaging scan was not performed in this case. This was because the pathological diagnosis of gossypiboma was confirmed preoperatively and the inclusion of metal in the mass could not be completely ruled out due to the lack of surgical records at that time. Magnetic resonance imaging findings of gossypiboma are generally reported to show a low signal on T1‐weighted and a high signal on T2‐weighted.^9^

There have been rare reports of preoperative diagnosis of gossypiboma observed using endoscopic ultrasound and histological examination using EUS‐FNA. Kato et al. first reported a case of intra‐abdominal gossypiboma, which was difficult to diagnose preoperatively using CT but was successfully diagnosed through transcolon puncture.^6^ To the best of our knowledge, our case represents the second reported instance of gastrointestinal puncture and the first report of transgastric puncture. Although the endoscopic ultrasound findings, in this case, were partially consistent with abdominal ultrasound findings previously reported in a retrospective imaging study, such as the presence of an intense post‐acoustic shadow, differentiation from a submucosal tumor of the gastrointestinal tract was difficult when the lesion was in contact with the gastrointestinal tract. In fact, EUS findings, in this case, showed that the mass was partially continuous with the muscularis propria of the stomach. In this case, intraoperative findings showed that the gossypiboma was firmly attached to the adjacent stomach, spleen, and transverse colon. This may explain why the intra‐abdominal gossypiboma was observed to be contiguous with the adjacent muscularis propria of the gastrointestinal tract on EUS. Despite CT findings being suggestive of gastrointestinal stromal tumor, gossypiboma was not considered in the differential diagnosis. Diagnosis by EUS may have been difficult due to the rarity of this condition and limited reports on its observation using EUS. This is the second report on gossypiboma observed in the gastrointestinal tract. As the number of cases observed using EUS increases in the future, it may be possible to diagnose the disease solely with endoscopy.

Although reports regarding the safety of puncturing an intra‐abdominal gossypiboma through the gastrointestinal tract are lacking, we did not observe any complications from puncture to surgical removal in this case. The interior of the mass in this case appeared to have remained sterile, and no evidence of infection was observed in the resected specimen. However, CT scans after the puncture revealed shrinkage of the mass and images of internal gas, suggesting the possibility of bacterial contamination from the puncture. Considering the risk of abscess formation, prompt surgical removal may be necessary after puncture depending on the puncture site.

This report highlights that tissue diagnosis using transgastric EUS‐FNA may be useful for the preoperative diagnosis of intra‐abdominal gossypiboma, which is difficult to diagnose preoperatively through imaging. In the case of puncture of gossypiboma, the use of a larger diameter puncture needle, such as the 19G needle used in this case, may facilitate the pathologist's diagnosis, since the collection of gauze fragments is necessary for needle biopsy. However, since puncture also carries the risk of causing infection to the mass, if a PAS such as the one seen in this case is found in a lesion that is suspected to be a gastrointestinal submucosal tumor, the possibility of gossypiboma should be considered and a consultation with a surgeon may be necessary.

## CONFLICT OF INTEREST STATEMENT

None.

## ETHICS STATEMENT

Not applicable.
